# Structural and Phylogenetic Analysis of *CXCR4* Protein Reveals New Insights into Its Role in Emerging and Re-Emerging Diseases in Mammals

**DOI:** 10.3390/vaccines11030671

**Published:** 2023-03-16

**Authors:** Fouzia Naheed, Rabia Mumtaz, Sana Shabbir, Arshad Jamil, Akhtar Rasool Asif, Abdur Rahman, Hafiz Ishfaq Ahmad, Muhammad Essa, Hammad Akhtar, Samy F. Mahmoud, Fatimah Othman Alghamdi, Hala Abdulrahman Al Amari, Jinping Chen

**Affiliations:** 1 Rural Health Centre Khayaban e Sir Syed, Rawalpindi 46000, Pakistan; 2 Basic Health Unit 6/1_L Renala Khurd District Okara, Rawalpindi 46000, Pakistan; 3 Government City Dispensary Hajveri Town, Faisalabad 38000, Pakistan; 4 Department of Plant Breeding and Genetics, Faculty of Agriculture, University of Agriculture, Dera Ismail Khan 29111, Pakistan; 5 Department of Animal Sciences, Sub-Campus, University of Veterinary and Animal Sciences, Lahore 35200, Pakistan; 6 College of Animal Science and Technology, Huazhong Agricultural University, Wuhan 430000, China; 7 Department of Animal Breeding and Genetics, Faculty of Veterinary and Animal Sciences, The Islamia University of Bahawalpur, Bahawalpur 63100, Pakistan; 8 Department of Gynecology, King Edward Medical University, Lahore 54000, Pakistan; 9 Medicine Department, Ghurki Trust Teaching Hospital, Jallo Morr, Lahore 54000, Pakistan; 10 Department of Biotechnology, College of Science, Taif University, P.O. Box 11099, Taif 21944, Saudi Arabia; 11 Bioengineering Institute, King Abdulaziz City for Science and Technology, P.O. Box 6086, Riyadh 11442, Saudi Arabia; 12 Guangdong Key Laboratory of Animal Conservation and Resource Utilization, Guangdong Public Laboratory of Wild Animal Conservation and Utilization, Institute of Zoology, Guangdong Academy of Sciences, Guangzhou 510260, China

**Keywords:** *CXCR4*, chemokines, immune system, diseases, phylogenetics

## Abstract

Chemokine receptor type 4 (*CXCR4*) is a G protein-coupled receptor that plays an essential role in immune system function and disease processes. Our study aims to conduct a comparative structural and phylogenetic analysis of the *CXCR4* protein to gain insights into its role in emerging and re-emerging diseases that impact the health of mammals. In this study, we analyzed the evolution of *CXCR4* genes across a wide range of mammalian species. The phylogenetic study showed species-specific evolutionary patterns. Our analysis revealed novel insights into the evolutionary history of *CXCR4*, including genetic changes that may have led to functional differences in the protein. This study revealed that the structural homologous human proteins and mammalian *CXCR4* shared many characteristics. We also examined the three-dimensional structure of *CXCR4* and its interactions with other molecules in the cell. Our findings provide new insights into the genomic landscape of *CXCR4* in the context of emerging and re-emerging diseases, which could inform the development of more effective treatments or prevention strategies. Overall, our study sheds light on the vital role of *CXCR4* in mammalian health and disease, highlighting its potential as a therapeutic target for various diseases impacting human and animal health. These findings provided insight into the study of human immunological disorders by indicating that Chemokines may have activities identical to or similar to those in humans and several mammalian species.

## 1. Introduction

Chemokines are a family of small proteins that play an essential role in the immune system by guiding the movement of immune cells to the site of infection or injury. They are secreted by various types of cells, including leukocytes, fibroblasts, endothelial cells, and epithelial cells [1]. Chemokines act as chemoattractants by binding to chemokine receptors on the surface of immune cells, thereby triggering a series of intracellular signaling events that result in cell migration [2]. Chemokine receptors are expressed on the surface of various immune cells, such as T cells, B cells, monocytes, and dendritic cells. They recognize and bind to specific chemokines, and the resulting signaling pathways direct the migration of immune cells to the site of infection or injury [3]. Chemokines and chemokine receptors are essential components of the immune system, and their dysregulation has been implicated in various diseases, including cancer, autoimmune disorders, and chronic inflammation. Therefore, targeting chemokines and chemokine receptors has emerged as a promising therapeutic strategy for treating these diseases [4].

Some examples of these chemokines are *CX3C*, *ACKR1*, *ACKR2*, *ACKR3*, and *ACKR4*, and these are the receptors of chemokine’s *CX3CR1* [5]. The chemokine receptor is thought to have a two-step binding mechanism that it employs to interact with the chemokine ligands that it recognizes: chemokine recognition site 1, or *CRS1*, is where the chemokine’s structured C-terminal component first attaches to the receptor’s N-terminus region and extracellular loops (ECLs), which then allows the chemokine’s unstructured N-terminus to target the receptor’s 7-TM helical bundle [5,6]. Chemokine receptors are becoming increasingly interesting as prospective therapeutic targets for treating inflammatory disorders and cancer due to their crucial function in the motility of cells [7]. Herpesviruses carry DNA that encodes receptors strikingly comparable to human chemokine receptors, such as *ORF74*, *BILF1*, and *US28*. This allows the herpesvirus to control the host’s chemokine receptor-mediated cell signaling networks. Consequently, it is feasible to consider these antiviral therapy targets as chemokine receptors [8]. *CXC* chemokine receptor 4, or *CXCR4*, is a member of the G protein-coupled receptor (*GPCR*) family of proteins. *GPCRs* are responsible for more than thirty percent of the pharmacological targets currently being pursued. Due to its involvement in the migration of hematopoietic stem cells, its role in dysregulating *CXCR4* in various human cancers, and HIV-1’s use of *CXCR4* to enter T cells, this receptor is becoming an increasingly important therapeutic target [9]. Only one treatment for *CXCR4* has been approved by the FDA (Mozobil, for hematopoietic stem cell mobilization), and many other treatments for this target are now under development for cancer and other conditions [10]. The crystal structures of class A *GPCR* superfamily members in active and inactive states provide a unique window into the structural underpinnings of ligand binding, G protein coupling, and *GPCR* activation via transmembrane (TM) helix rearrangements. When the *GPCR* is active, helices V and VI, and III and III under some conditions, undergo significant conformational changes [11]. On the other hand, static representations have not successfully explained the residue-level processes behind the dynamic helical modifications responsible for *GPCR* signal transduction. In addition, the structures of the *GPCR’s CXCR4* and the vast majority of other *GPCRs* have only been solved in their inactive states [12]. Extensive mutagenesis research on *GPCRs* in general (describing over 8000 mutations (gpcrdb.org) and *CXCR4* in particular (covering 81 mainly extracellular residues of 352 total) [13] has led to the discovery of specific residues that play a key role in receptor signaling. Even though many of the key residues and motifs have been described individually, the intramolecular signal transmission chain is still a mystery [14]. It has been demonstrated that the signaling of *CXCR4* plays an important part in the bone marrow niche. In this environment, it governs the survival of HPCs and their movement and activation [15]. Due to its several roles in the immune system, the *CXCR4* axis is a signaling circuit that has been extensively investigated and conserved [16]. Unfortunately, we lack a thorough grasp of their fundamental structure and function, so attempts to use this species as an efficient immunological model are severely hindered [17]. According to research on the chemokine family’s evolution [18], chemokines descended from a single ancestor 650 million years ago and have progressed through a series of duplication events [19]. The chemokine family has primarily evolved due to tandem gene duplication events, according to studies examining chemokines’ genomic organization across mammalian species’ genomes [19]. Researchers have carried out these studies. The formation of this multigene protein family was also influenced by many other evolutionary processes [20], such as the birth and death of genes, the insertion and deletion of nucleotides, and the change in nucleotides. There has been a delay in tracing evolution’s structural and functional consequences concerning these molecular messengers, despite several reports having uncovered varied evolutionary perspectives about them [21]. *CXCR4* chemokines, which are part of the family of neutrophil-activating chemokines, were the ones we went with so that we might glean some information regarding the evolutionary characteristics of neutrophil-activating chemokines (NACs) [22]. To analyze Growth-Related Oncogene (GRO) genes over a wide range of mammalian species, we used phylogeny, selection, and substitution rate analyses, as well as conservation scores, nucleotide alterations, and electrostatic surface potentials [23]. *CXCR4* is a protein that is found in many different taxa, including humans, mice, rats, and dogs. The protein is a G protein-coupled receptor that is involved in many different physiological processes, including immune function, development, and cell migration [24]. The primary structure of *CXCR4* is similar across different taxa, with the human and mouse *CXCR4* proteins sharing 93% amino acid identity. The protein consists of 352 amino acids and has a predicted molecular weight of approximately 40 kDa [25]. However, there are some amino acid differences among the taxa. For example, the human *CXCR4* protein has a histidine residue at position 281, while the mouse *CXCR4* protein has a tyrosine residue at this position. In addition, the dog *CXCR4* protein has a serine residue at position 336, while the human and mouse proteins have an alanine residue at this position [26]. Despite these differences, the overall structure and function of *CXCR4* is conserved across different taxa. This makes it a valuable target for drug development and other therapeutic interventions [27].

Our study aims to conduct a comparative structural and phylogenetic analysis of the *CXCR4* protein to gain insights into its role in emerging and re-emerging diseases that impact the health of mammals. By conducting comparative structural and phylogenetic analyses, the manuscript offers new insights into the function of *CXCR4* in disease susceptibility or resistance, potentially leading to new treatments or prevention strategies. This aims to contribute to the current understanding of *CXCR4* and its potential as a therapeutic target for diseases impacting mammalian health.

## 2. Materials and Methods

### 2.1. Retrieval of CXCR4 Sequences

To retrieve the nucleotide and amino acid sequences of the *CXCR4* gene from a specific organism, you can use publicly available databases such as NCBI, Ensembl, or UniProt [28]. We used the NCBI Public Archive (https://www.ncbi.nlm.nih.gov/, accessed on 5 March 2023) [29] to get the human *CXCR4* gene family’s amino-acid sequence and the PDB Public Archive (http://www.rcsb.org/public/pdb/, accessed on 5 March 2023) to find the protein’s crystal structure [30]. We used BLAST (Basic Local Alignment Search Tool) to retrieve *CXCR4* sequences from mammalian species. These retrieved sequences were used to perform various types of analysis, such as phylogenetic, structural, and functional analyses.

### 2.2. Phylogenetic Analysis of the CXCR4 Gene

We obtained *CXCR4* amino acid sequences from various mammalian species, including primates, rodents, carnivores, and ungulates, from publicly available databases such as NCBI and UniProt [29]. These sequences were aligned using Clustal Omega software to create a multiple-sequence alignment. We used the maximum likelihood method for phylogenetic tree construction, which we performed using MEGA (Molecular Evolutionary Genetics Analysis) software version 10.0.5 [30]. We used the neighbor-joining method for initial tree construction and assessed the tree topology using the maximum likelihood method with the Whelan and Goldman (WAG) substitution model [31]. We also performed 1000 bootstrap replications to assess the robustness of the tree topology. TreeBeST generated the species tree as a reference for assessing gene trees or other phylogenetic trees [32]. This is because the species tree represents the true evolutionary relationships among the species under study.

In contrast, gene trees may be affected by incomplete lineage sorting, duplication, and loss [32]. When analyzing the phylogenetic relationships of a specific gene or protein, it is important to compare the gene tree with the species tree to ensure that the gene has evolved in a manner consistent with the known evolutionary history of the species. The species tree can be used to test hypotheses regarding the gene’s evolutionary history and to infer the direction and frequency of evolutionary events such as gene duplication, gene loss, and functional divergence [33].

The gene gain and loss tree was constructed using a maximum likelihood approach [34]. The phylogenetic analysis was performed using the software RAxML, which implements a fast and efficient algorithm for the maximum likelihood estimation of phylogenetic trees [35]. To construct the tree, we first aligned the protein sequences of interest using the MUSCLE algorithm [36]. We then used RAxML to estimate the phylogenetic tree based on the multiple sequence alignment. We selected the best-fit substitution model for the analysis using the Akaike Information Criterion (AIC) [37]. The bootstrap method was used to assess the robustness of the inferred tree. Specifically, we performed 1000 bootstrap replicates to estimate the support for each tree branch [38]. The gene gain and loss tree was mapped onto the tree using the Ensembl database to obtain the gene family information. The HKY (Hasegawa–Kishino–Yano) model was used to estimate the evolutionary distances between gene family members [39]. The HKY model is a widely used phylogenetic model that considers the unequal nucleotide substitution rates and the probabilities of transitional and transversional mutations [40]. This model was used to estimate the nucleotide substitution rate and infer the phylogenetic relationships between the gene family members. The gene gain and loss events were then mapped onto the tree to provide insights into the evolutionary history of the gene family [41].

### 2.3. Prediction and Validation of Human CXCR4 Structure

Human *CXCR4* protein crystal structure was determined using the Protein Data Bank. Therefore, the predicted 3D structures of *CXCR4* were successfully modeled using homology modeling approaches. Phyre2 [42], the Swiss model [43], and I-TASSER [44], which operates numerous threading approaches after recognizing the template from PDB, were among the software programs we utilized to build or anticipate the precise 3D modeled structure of the target proteins. The target proteins were reduced to the smallest possible sizes using the conjugate gradient approach and the Amber force field in UCSF Chimera 1.10.1. [45]. There were still obstacles when it came to validating predicted protein structures. To carry out our inquiry, we used several different protein structure validation approaches, one of which was called protein structure analysis. Our investigation aimed to identify defects in the experimental and theoretical protein structure generated using 3D structure modeling [46]. The ProSA software package aims to validate the atomic structure coordinates anticipated by the ProSA program, and the z-score value is used to evaluate the findings [47].

### 2.4. Disordered Analysis of Human CXCR4 Proteins

Our goal was to determine which protein segment or unstructured sections were present in the human and mouse *CXCR4* proteins after doing 3D homology modeling of both proteins [48]. This area is thought to contribute to protein instability, leading to the development of pathogenic diseases. We used Cspritz version 1.2 (http://protein.bio.unipd.it/cspritz/, accessed on 5 March 2023) to accurately forecast the locations of unstructured protein segments of amino acid residues, and we could do so with high accuracy [49].

### 2.5. Analysis of Human CXCR4 Protein Ligands and Domain

In proteomics, it is essential to comprehend the structural properties of a protein’s functional unit, as this helps determine its function. Therefore, the protein–ligand interaction, ligand-binding residue, ligand-binding areas, and domains of *CXCR4* were predicted utilizing a wide range of bioinformatics techniques. Additionally, the protein ligands were categorized based on how similarly their roles functioned. The ligand-binding residues in the protein structures were predicted using a template-based, robust 3D modeling online tool [50]. It is well-known for its high-quality structural modeling output, which may be shared across multiple targets using a single remote template. We employed a few other programs, including the COACH server [51], to double-check and verify the accuracy of the projected result. I-TASSER was utilized to ascertain the number of binding sites as well as the locations of those binding sites.

Additionally, I-TASSER was utilized to ascertain the number of binding sites and the positions of those binding sites [44]. COACH is a system that utilizes a meta-server to predict ligand binding targets. This strategy uses two comparison methodologies, TM-SITE [44] and S-SITE. In these methodologies, the BioLiP protein function database is consulted to locate ligand binding sites, which are then used in the formulation of new ligand binding sites [52]. We could better know the ligand-binding surface on a non-bound form of a freely occurring protein structure by using the ligand-binding prediction tools available on the FTSite server [53]. Its accuracy is comparable to that of experimental findings in terms of its precision [54]. The prediction method will likely be used to map protein areas; this would explain why it is called “protein region mapping” [55]. Ligand module clustering was performed using the web server LPIcom (Singh et al., 2016; http://crdd.osdd.net/raghava/lpicom, accessed on 5 March 2023) to learn more about the relationships between ligands and amino acids in the target protein. It was possible to predict the interaction between amino acid residues and ligands by categorizing them using the LPIcom web server following their predicted interaction and binding motif for a certain ligand [56,57].

### 2.6. Protein Interactions and Co-Expression Analysis

To determine the functional connections and information flow networks between *CXCR4* and its surrounding genes and other proteins in humans and mice, an investigation into the protein–protein interactions that occur between *CXCR4* and its surrounding genes and other proteins was necessary. Various physiological conditions, including protein–protein interactions, have been found to affect several biological processes. The interaction analysis was carried out using the STRING program [58], and the visualization was performed using the commercial Cytoscape software [59]. The STRING tool takes into account both the functional and physical relationships that exist between proteins [60].

### 2.7. Consensus Sequence and Secondary Structure Prediction

To compare *CXCR4*’s biochemical and structural alignments, we used the freely available application ENDscript 2 [61]. Utilizing this approach, we gained a better understanding of the structural alignment of *CXCR4* [61]. The structure recognition capability of the web tool extends from the most fundamental to the most complicated levels, or from the primary to the quaternary levels. In addition, it uses the Protein Data Bank (PDB) [29] as the input format. It separates the results into various outcomes, all viewed via a selection of different structure interface tools. The secondary structure of each target protein was also determined with the assistance of the web-based server PSIPRED version 3.3 [62]. PSIPRED is a web-based technology combining protein sequence and structural analysis into a single platform. Initially, the developer uses protein sequence input data to conduct PSI-BLAST searches on the findings [63].

## 3. Results

Our study aimed to investigate the evolutionary history and potential functional roles of *CXCR4*. This chemokine receptor protein plays a critical role in immune system function and is implicated in various diseases. To achieve this, we conducted a comparative structural and phylogenetic analysis of *CXCR4* sequences from 30 mammalian species, including emerging and re-emerging disease hosts. Our results revealed several key insights into the evolution and potential functional roles of *CXCR4*. First, we found that *CXCR4* is highly conserved across mammalian species, with most amino acid residues being conserved across all sequences. However, we also identified several positive selection sites, suggesting that *CXCR4* has undergone adaptive evolution in response to various selective pressures. Second, our phylogenetic analysis revealed that *CXCR4* has a complex evolutionary history, with multiple gene duplication and loss events occurring throughout mammalian evolution. Despite these events, we reconstructed the evolutionary relationships among *CXCR4* sequences from different species and identified several distinct clades of *CXCR4* sequences with potential functional significance. Finally, we conducted a structural analysis of *CXCR4* based on its crystal structure, which allowed us to identify key functional domains and amino acid residues that are conserved across different species. We also identified several potential functional sites under positive selection or evolving convergently in different lineages.

### 3.1. Evolutionary Analysis of the CXCR4 Gene

**Figure 1:**
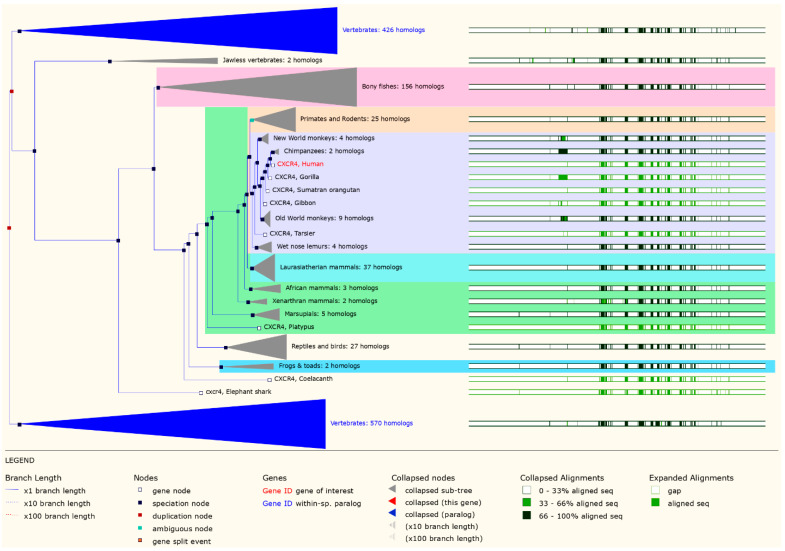
The phylogenetic tree with the highest likelihood represents the evolution of the *CXCR4* gene. The red boxes represent gene duplication events, and the blue boxes represent speciation events. In the alignment of domains, the green and black bars represent the conserved and variable regions of the *CXCR4* protein, respectively.

In our study, we constructed gene trees for *CXCR4* of mammalian species using maximum likelihood methods. Gene trees show the evolutionary path taken by gene families that descended from a single common ancestor. Our analysis revealed that *CXCR4* has a complex evolutionary history, with multiple gene duplication and loss events occurring throughout mammalian evolution. Despite these events, we reconstructed the evolutionary relationships among *CXCR4* sequences from different species and identified several distinct clades of *CXCR4* sequences with potential functional significance. In particular, we found that *CXCR4* sequences from primates and rodents form distinct clades, suggesting that these two groups have undergone different evolutionary trajectories regarding *CXCR4* evolution (Figure 1). Our gene tree analysis also revealed several instances of convergent evolution, where amino acid changes occurred independently in different lineages. These concurrent changes may indicate functional adaptation to similar selective pressures in different environments or hosts. The tree is presented with branch lengths proportional to the amount of evolutionary change that has occurred. Bootstrap values are indicated at the nodes to assess the support for each branching pattern. The tree is color-coded to indicate the major mammalian clades, including primates, rodents, carnivores, and ungulates. In addition to the tree, we also show an alignment of the *CXCR4* protein sequences from representative species, highlighting the key domains and motifs within the protein.

**Figure 2:**
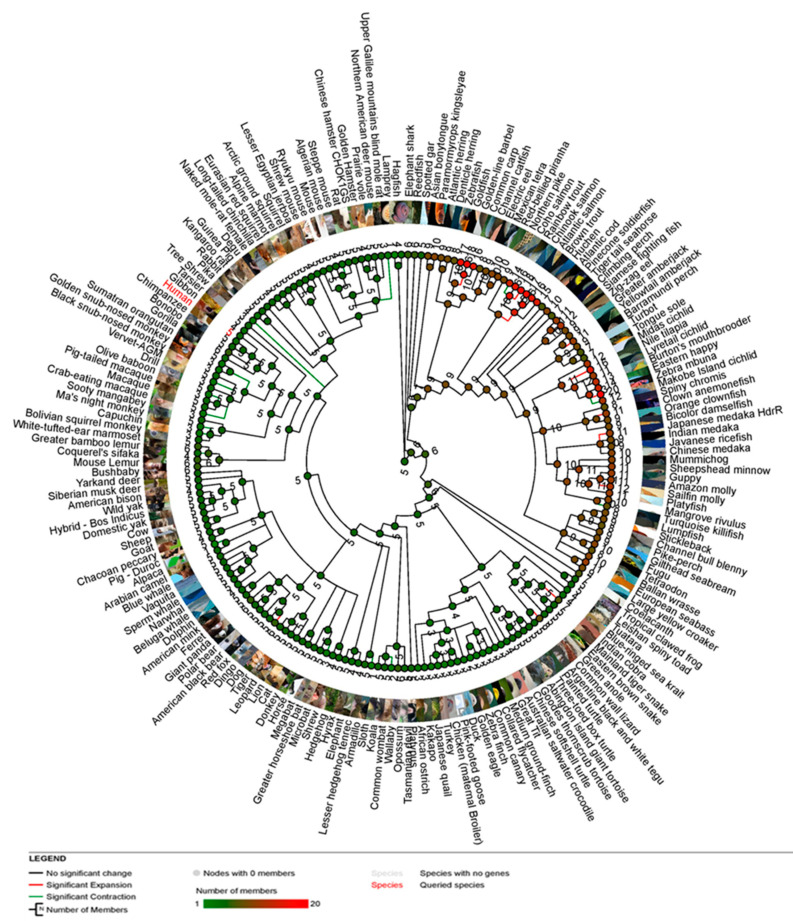
The evolutionary history of the *CXCR4* gene is shown by the gene gain/loss tree, which highlights the genes that have been added and subtracted over time.

The gene gain and loss tree constructed using the Ensembl database and the HKY model revealed the evolutionary history of the *CXCR4* gene family. The tree showed multiple gene gains and loss events, with some lineages experiencing expansions and contractions at different times. Specifically, the gene family underwent a series of gene duplications and losses throughout its evolution, with some duplications occurring in specific lineages such as primates and rodents. The tree also showed that the *CXCR4* gene family has a conserved domain structure across different species, suggesting functional protein conservation. Overall, the gene gain and loss tree provided insights into the evolutionary dynamics of the *CXCR4* gene family and its functional significance in different organisms (Figure 2). The resulting gene gain and loss tree provide insights into the evolutionary history of the *CXCR4* gene family and how it has evolved in different lineages over time. By identifying the events of gene duplication and loss, we can gain insights into the functional diversification of the *CXCR4* gene family and how it has contributed to the emergence of different *CXCR4*-related diseases in mammals. The phylogenetic study of the *CXCR4* protein from several mammalian species revealed that humans are clustered with gibbons, chimpanzees, bonobos, and gorillas (Figure 2).

Interestingly, the signal peptide region was the only spot where amino acid diversity was observed. The major secreted region of human *CXCR4* was identical to that of mammalian CXCL12, suggesting that the two proteins share the same evolutionary origin (Figure 1). Humans, gibbons, chimpanzees, bonobos, and gorillas were grouped together on the *CXCR4* phylogenetic tree (Figure 2). The clustering of humans with these different species in a phylogenetic tree reflects their evolutionary relationships and the degree of genetic similarity between their genomes. The grouping of humans with primates such as gibbons, chimpanzees, bonobos, and gorillas reflects their close evolutionary relationships and the fact that they share a common ancestor relatively recently in geological time (Figure 2).

### 3.2. Structural Analysis of CXCR4 Protein

**Figure 3:**
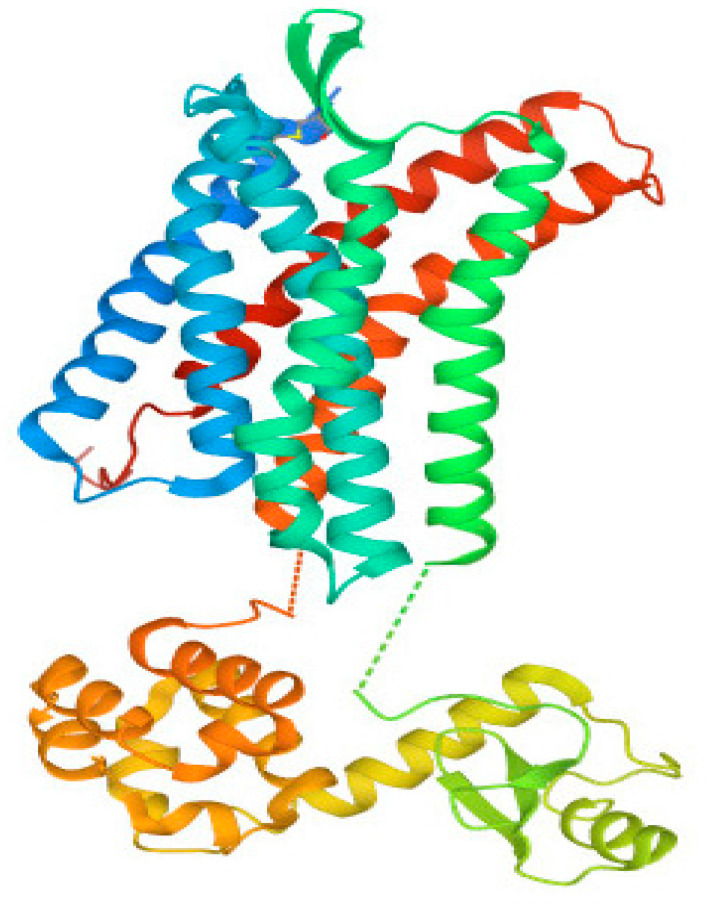

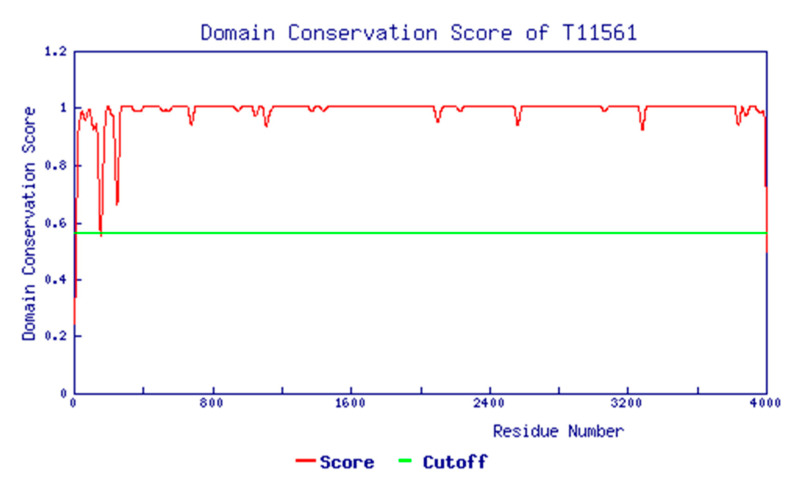
The crystal structures of human proteins and conservation analyses were used to simulate the homology of the *CXCR4* structure. The evolutionary rate of a site is reflected in the conservation score at that location.

**Figure 4:**
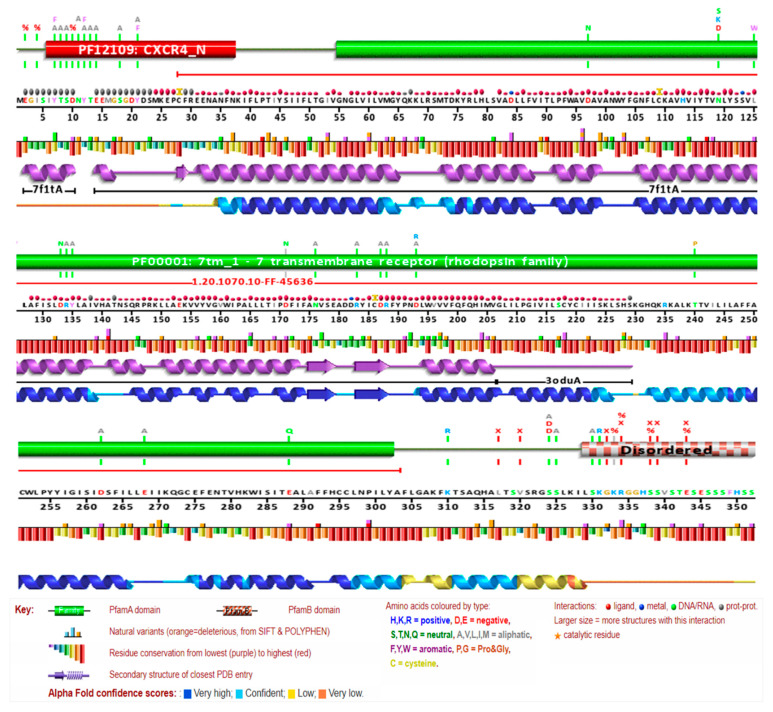
Prediction of the secondary structure of *CXCR4* protein. Conserved residues are represented by letters, and helices in the tertiary structure are shown in different colors. Red bar points out a protein’s three main structural elements: a β-sheet, a coil, and an α helix. The human *CXCR4* protein is depicted in the upper panel.

Mammalian *CXCR4* proteins, which have a conserved amino acid sequence, can have their 3D protein structures predicted using homology modeling (HM), accomplished using crystal structures of human *CXCR4* proteins. The structure of the *CXCR4* protein found in humans consisted of two αhelixes, three anti-parallel β-sheets, and four loops. This structure is quite similar to *CXCR4* proteins found in other animals. It was found that the human *CXCR4* protein shares the *CXCR4*-binding sequence found in human *CXCL12* (Figure 3). Two anti-parallel βsheets, three extracellular loops, and seven transmembrane α-helices were found in the three-dimensional structures of both mouse and human *CXCR4* proteins (Figure 4). The active amino acid residues of mammalian *CXCR4* are in the protein’s active domain. It was shown that the *CXCR4* protein sequence was conserved across multiple mammalian species, including humans. However, the semi-conserved amino acid residues in the structure of the *CXCR4* protein, which is found in humans and other mammals, are not organized into any functional domains. These findings suggested that the *CXCR4* proteins in other mammalian species have a binding relationship analogous to the one in humans. In addition, the secondary structure of the *CXCR4* protein has been analyzed with PSIRED, and the results have shown that the secondary structure contains various coils, helices, and strands. This indicates that the secondary structure is highly complex (Figure 4). To examine and forecast the secondary structure of the human *CXCR4* protein, the 3D protein structure modeling capabilities of the web server RaptorX were put to use. A blue arrow, the coil by a yellow arrow, and the α helix by a red arrow, represent the β-helix, respectively. Within three subunits, residues are depicted in a conformation that rotates them away from the active site (Figure 4).

The amino acid positional behavior at catalytic sites or elsewhere is necessary for protein interactions, which underpin a wide range of fundamental biological processes. This means that some amino acids are more easily recognized and stable than those that comprise a given protein sequence. To a greater extent, mutations resulting from changes in amino acids at sites where they have been highly conserved throughout evolution are predicted to be deleterious. The conservation rate, important for the in-depth analysis of the anticipated effects of the high-risk SNPs, was calculated by applying the ConSurf algorithm to the amino acids of the human *CXCR4* protein and seeing it from an evolutionary perspective. An elaborate neural network can reliably predict the secondary structure of a given molecule with an estimated average accuracy of greater than 72%. Neuronal networks with a 17-node window predict the secondary structure and solvent accessibility. The *CXCR4* protein contains seven transmembrane helices (TM1–TM7) that span the plasma membrane. The N-terminus of the protein is located extracellularly, while the C-terminus is located intracellularly. The intracellular region also contains several domains, including the G protein-binding domain, which activates downstream signaling pathways upon ligand binding.

To calculate the average fraction of the pairwise sequence identity of the *CXCR4* protein, we aligned all of the *CXCR4* protein sequences from different species and calculated the percentage identity between each pair of sequences. The average identity was calculated by summing the pairwise identities and dividing by the total number of pairwise comparisons. A BLAST search was carried out against UniProtKB/SwissProt, and the results were then aligned with MaxHom to acquire homology information. The input for the network is the multiple sequence alignment produced as a result of the analysis. A pairwise sequence alignment identity matrix is a square matrix that represents the percentage of identical residues between two sequences. The matrix is symmetric, with each element representing the identity between a pair of residues in the two sequences. The diagonal elements represent the identity between identical residues in the two sequences, while the off-diagonal elements represent the identity between non-identical residues.

**Figure 5:**
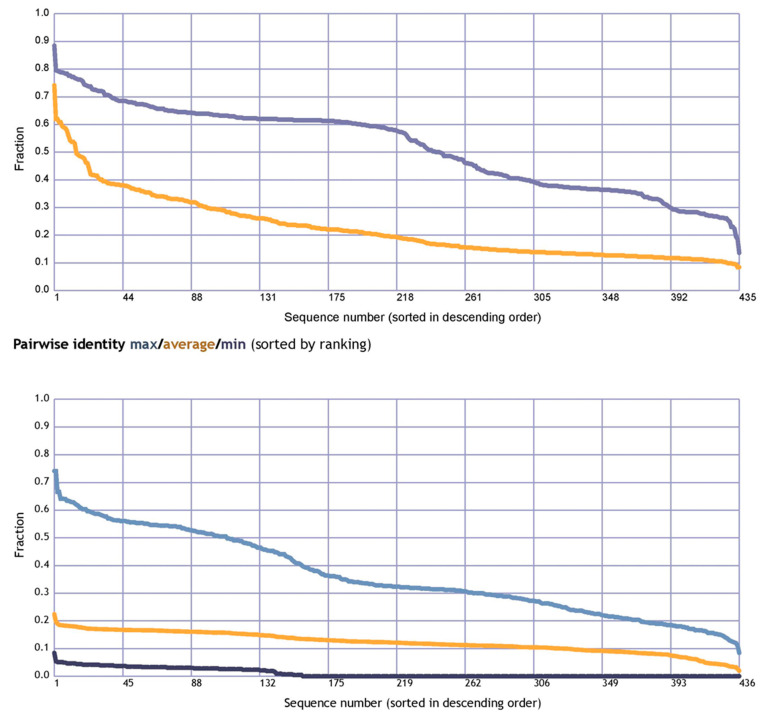
Utilizing evolutionary data from multiple sequence alignments, RePROF predicted solvent accessibility and secondary structure elements.

**Figure 6:**
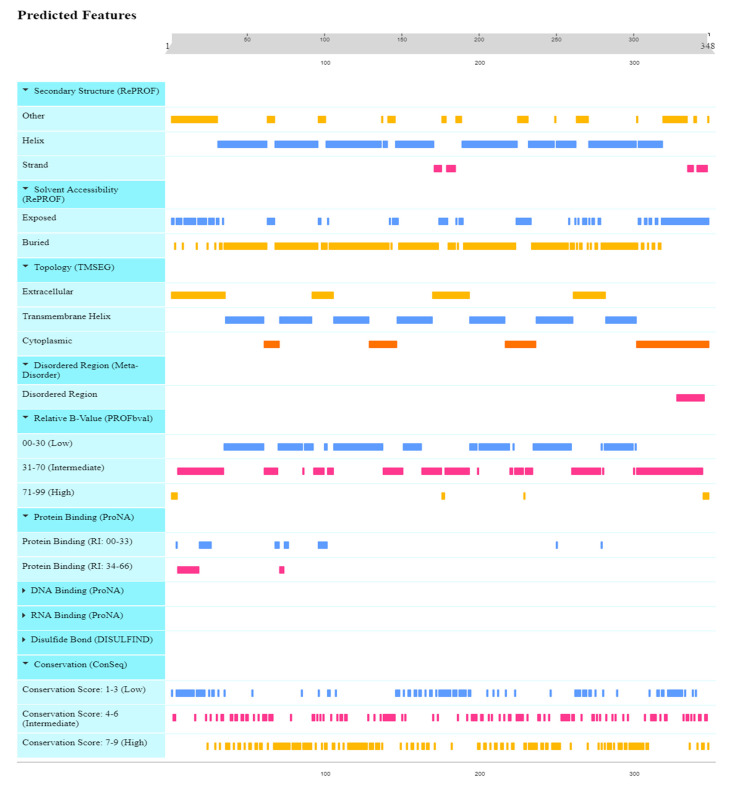
Structural features of the *CXCR4* protein (motifs, interaction sites, DNA, RNA binding, topology, and conservation) were predicted using the Rost Lab’s Reprof secondary structure predictor and accessibility predictor. An alignment of human *CXCR4* sequences was used to make the prediction.

When the dependability indices have high values, this indicates that the forecasts are more. A 70% anticipated accuracy does not necessarily mean that 70% of your protein’s predicted residues are accurate. Instead, this value is derived by taking the average of many proteins whose behavior is difficult to anticipate. Therefore, the forecast accuracy for your protein can be higher than 80% or lower than 60%. (Figure 5). The pairwise sequence alignment identity matrix was used to compare the similarity between different *CXCR4* protein sequences. The higher the identity between two sequences, the more similar they are likely to be. However, it is important to note that a high sequence identity does not necessarily mean the two sequences have the same function or structure. Other factors, such as sequence length, sequence conservation, and amino acid substitutions, can also play a role in determining the similarity between sequences. The RePROF method has been used to predict the secondary structure of *CXCR4* proteins. RePROF is a computational method that combines multiple sequence alignment and a profile–profile comparison to predict the protein secondary structure with high accuracy. The results of the RePROF analysis showed that *CXCR4* proteins contain seven transmembrane helices, as expected for a G protein-coupled receptor. In addition, the analysis identified the location of the intracellular loops and extracellular loops within the protein structure. The RePROF method predicted solvent accessibility and secondary structure elements. Solvent accessibility refers to the extent to which a residue is exposed to solvent and is an essential feature for understanding protein folding, stability, and interactions with other molecules (Figure 6). The results of the RePROF analysis showed that the predicted solvent accessibility of *CXCR4* residues correlated well with their experimentally determined values.

Moreover, the analysis identified several residues highly exposed to solvent and may be involved in protein–protein interactions or binding to ligands. Overall, the results of the RePROF analysis provide important insights into the secondary structure of *CXCR4* proteins, which is essential for understanding their function and interactions with other proteins. The accuracy of the RePROF method suggests its potential usefulness for predicting the secondary structure of other proteins as well. (Figure 6). The topology of *CXCR4* refers to the arrangement of its transmembrane helices and the orientation of the protein in the membrane. *CXCR4* has a classic GPCR topology, with the N-terminus located extracellularly and the C-terminus located intracellularly. The seven transmembrane helices are arranged in a helical bundle, with the extracellular and intracellular loops connecting the helices (Figure 6).

### 3.3. Functional Analysis

**Figure 7:**
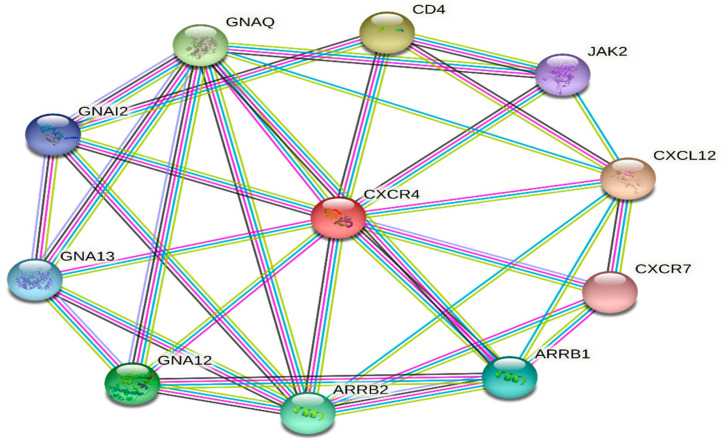
*CXCR4* protein–protein interaction analysis. The length of an interaction between two proteins is one way to measure how far apart the proteins are. The nodes represent the proteins, and the edges represent their interactions. The size of the nodes corresponds to the degree of connectivity, with larger nodes representing more highly connected proteins. The nodes’ colors indicate the proteins’ functional categories.

The functional analysis uncovered ten putative interacting partners for *CXCR4* in the protein interaction network as determined via the STRING analysis (Figure 7). The query protein has eleven proteins that interact most closely with *CXCR4*, including proteins involved in immunomodulation, organogenesis, hematopoiesis, and activities that are disrupted in cerebellar neuron migration. According to the findings of the STRING database study, the protein–protein interaction (PPI) network is made up of 11 nodes that are connected by a total of 27 distinct edges. The predicted number of edges was 17, and the average degree score for a node was 4.91. This indicates that each node had at least 4.91 other nodes with whom it interacted. The PPI enrichment *p*-value and the average local clustering coefficient came in at 0.793. The PPI enrichment *p*-value was observed to be 0.0113. *CXCR4* was found to have a very high confidence level in its ability to interact with ten other proteins, as demonstrated by protein–protein interaction (PPI) networks. This indicates that the *CXCR4* proteins have more connections among themselves than would be predicted for a random group of proteins taken from the genome that were the same size and had the same degree of distribution. Enrichment of this kind suggests that the proteins form a group that shares at least some biological connections (Table 1).

## 4. Discussion

The structural and phylogenetic analysis of the *CXCR4* protein has provided new insights into its biological functions and role in emerging and re-emerging diseases in mammals. The *CXCR4* protein is a G protein-coupled receptor that plays a crucial role in various physiological processes such as immune response, hematopoiesis, and stem cell migration. Analysis of the *CXCR4* crystal structure has improved our understanding of how *CXCR4* interacts with its ligands, such as the chemokine *CXCL12*, and how ligand binding induces conformational changes in the receptor, resulting in downstream signaling. However, the structural and phylogenetic analysis of *CXCR4* has provided new insights into its function in mammals and its role in various diseases. The information obtained from these analyses can guide the development of new therapeutics for diseases that involve *CXCR4*, particularly those caused by viral infections [64]. Pathological alterations in cancer cells, such as metastasis development and aberrant blood vessel expansion, may be facilitated by *CXCR4*-mediated communication. As the co-receptor that directs the HIV into cells, *CXCR4* also plays a crucial role in HIV infection [65]. The phylogenetic study of the *CXCR4* protein from several mammalian species revealed that humans were first clustered with gibbons and gorillas and then secondly with mice and rats (Figure 2). It is interesting to note that the signal peptide area was the only place where different amino acids were seen. The main part of the human *CXCR4* protein that is secreted is the same as the part of the mammalian *CXCL12* protein that is secreted (Figure 1). According to the findings of studies on the evolution of the chemokine family, chemokines descended from a single ancestor approximately 650 million years ago, went through a series of duplication events, and are still evolving [66]. Based on studies of the syntenic organization of chemokines across the genomes of several mammals, it has been speculated that tandem gene duplication is predominantly responsible for the expansion of the chemokine family. Various evolutionary events, such as the emergence and disappearance of genes, insertion and deletion of nucleotides, and modification of nucleotides, among many others, have influenced this multigene protein family [67]. *CXCR4* is a member of the superfamily of seven transmembrane G-protein coupled receptors structurally related to chemokine receptors (GPCRs). Structural analysis of *CXCR4* has revealed several important features of the protein, including the arrangement of its transmembrane helices and the location of its ligand-binding sites [68]. The extracellular domain of *CXCR4* contains several conserved regions, including the N-terminus and the second extracellular loop, which are critical for ligand binding. The intracellular domain of *CXCR4* contains several domains, including the G protein-binding domain, which is responsible for activating downstream signaling pathways upon ligand binding. Structural analysis revealed that *CXCR4* could form homodimers and heterodimers with other GPCRs, which may play a role in its function in various diseases [26].

The GPCRs are signaling molecules triggered by small ligands that can be either promiscuous or selective. When triggered by an agonist, GPCRs undergo rapid phosphorylation within the C-tail and the third intracellular loop [69]. *CXCR4* has a total of 21 possible phosphorylation sites. Chemokines, which are small low-molecular weight proteins, mediate various cellular processes, such as development, leukocyte trafficking, angiogenesis, and immunological response, by activating and signaling through *CCR5* and *CXCR4* [70]. *CXCR4*’s structure revealed the hallmark core of GPCRs, a cluster of seven α helices that traverse the cell membrane in a crisscrossing fashion. These are linked together by a sequence of loops visible on both sides of the membrane. These loops are responsible for a significant portion of the labor involved in identifying the chemokine and transmitting the signal inside the cell. There is a cup-shaped depression on the exterior of the molecule that functions as the binding site [71]. To further understand how the chemokine binds, crystal structures were produced with a long cyclic peptide coupled in the active site (top, from PDB entry 3oe0) and with a tiny inhibitor bound (bottom, from PDB entry 3odu), offering a starting point for the design of anti-HIV medicines [72]. It was determined that the human *CXCR4* protein also contains the *CXCR4*-binding region found in the human *CXCL12* protein (Figure 3). The three-dimensional structures of both mouse and human *CXCR4* proteins revealed the presence of seven transmembrane α-helices, two anti-parallel β-sheets, and three extracellular loops (Figure 4). The functional domain of *CXCR4* in mammals contains amino acid residues related to their biological functions. It was shown that the *CXCR4* protein sequence was conserved across multiple mammalian species, including humans. The semi-conserved amino acid residues do not change. Phylogenetic analysis of *CXCR4* has revealed that the protein is highly conserved across species, with a sequence identity of over 95% between humans and mice. The conserved regions are primarily located in the transmembrane helices and the ligand-binding sites, indicating the importance of these regions in the function of the protein. However, there are also several non-conserved regions in *CXCR4* that may play a role in its function in different species or in different diseases (Figure 4).

It has been demonstrated that the amino terminus of *CXCR2* and the second extracellular loop are essential for ligand identification and receptor activation. However, a negatively charged residue, Asp199, was found in a previous study of the *EC2* of *CXCR2* to be critical for controlling the rate of receptor internalization [73]. Additional research has revealed that the amino-terminal Asp9 of *CXCR2* can be mutated into a constitutively active form through single mutations (such as *D9K* and *D9R*). These findings imply that charged residues may influence the stability and activation of *GPCRs’* second extracellular loop [72].

Although the process by which ligands attach to receptors can vary, there are certain similarities in how ligands and receptors interact despite these differences. Within the transmembrane areas, small ligands such as photons, biogenic amines, and nucleosides bind, whereas big molecules such as peptides and proteins bind to the extracellular loops of the membrane [74]. Peptide ligands demonstrate a direct connection between the amino terminus and the extracellular loops. On the other hand, some peptide ligands can interact with both transmembrane domains and extracellular loops [75]. Disordered analysis of human *CXCR4* proteins has revealed the presence of intrinsically disordered regions (IDRs) within the protein structure. IDRs are protein regions that do not adopt a fixed tertiary structure but exist in a disordered state [76]. These regions are typically rich in polar and charged residues and play important roles in protein–protein interactions and signaling. Several studies have identified IDRs within the *CXCR4* protein, particularly in the intracellular loops and the C-terminal tail [77]. These regions are known to interact with intracellular signaling molecules, such as G proteins and beta-arrestins, and are essential for *CXCR4*-mediated signaling.

Moreover, mutations or deletions within the IDRs of *CXCR4* have been associated with various diseases, including cancer and HIV infection [78]. These mutations can disrupt protein–protein interactions and alter *CXCR4* signaling, leading to pathological consequences. The disordered analysis of *CXCR4* provides important insights into this protein’s structural and functional properties, particularly in its interactions with other proteins and downstream signaling pathways [79]. Understanding the role of IDRs within *CXCR4* may have implications for developing new therapeutic strategies for diseases associated with *CXCR4* dysfunction.

The neurological and immune systems depend on the intricate interactions between the various cell types that make up the system to provide highly specialized responses to different environmental stimuli. This capability partly develops from experiential patterning and necessitates that information be securely retained as memory while simultaneously maintaining plasticity or producing adaptive responses to novel stimuli. There is a possibility that the molecular solutions to these criteria originated in the neurological system and were later appropriated by the immune system to enable adaptive immunity [1].

Immune and neurological system development are both impacted similarly by *CXCL12* and *CXCR4*. In addition to the formation of differentiated functions, these effects also control the proliferation and survival of progenitor cells. Following the germinal phase, *CXCL12* regulates plasticity in both systems. It accomplishes this by affecting T and plasma cell memory storage and responsiveness to novel stimuli. It also impacts synaptic transmission in the CNS, especially in areas where learning is strongly correlated. All these processes can be viewed as homeostatic roles that guarantee cell location, identification, and functionality.

Functional analysis of the interacting proteins showed they were involved in various cellular processes, including immune response, signal transduction, and cell adhesion. Interestingly, several interacting proteins were also implicated in cancer progression, suggesting that the protein–protein interaction network involving *CXCR4* may be relevant to cancer biology [80]. Furthermore, the network analysis identified several hub proteins highly connected to other proteins in the network. These hub proteins may play critical roles in regulating the *CXCR4* network and represent potential targets for therapeutic intervention [81]. According to the findings of the STRING database study, the protein–protein interaction (PPI) network is made up of 11 nodes that are connected by a total of 27 distinct edges. The predicted number of edges was 17, and the average degree score for a node was 4.91. This indicates that each node had at least 4.91 other nodes with whom it interacted. The PPI enrichment *p*-value and the average local clustering coefficient came in at 0.793. The PPI enrichment *p*-value was observed to be 0.0113. *CXCR4* had a very high confidence level in its ability to interact with ten other proteins, as demonstrated by protein–protein interaction (PPI) networks. The structural and phylogenetic analysis of the *CXCR4* protein has provided significant insights into its biological functions and role in various diseases. By examining the protein’s structure and evolutionary relationships across different mammalian species, we have gained a better understanding of its interactions with other proteins and ligands and its potential as a therapeutic target for various diseases. Overall, the structural and phylogenetic analysis of the *CXCR4* protein has shed new light on its biological functions and role in various diseases, providing a basis for further research and development of targeted therapeutics.

## 5. Conclusions

In conclusion, our study provides new insights into the role of *CXCR4* in emerging and re-emerging diseases that impact the health of mammals. Our comparative structural and phylogenetic analysis has identified novel features and variations in *CXCR4* that may have functional implications in different species and disease contexts. Moreover, our analysis of the evolutionary history of *CXCR4* has revealed genetic changes that may have contributed to functional differences in the protein across different species, potentially explaining variations in disease susceptibility and resistance. Overall, our study provides a comprehensive understanding of the genomic landscape of *CXCR4* in the context of emerging and re-emerging diseases impacting mammalian health and suggests new avenues for research into the pathogenesis and treatment of these conditions. Our findings have important implications for developing targeted and personalized therapies for human and animal diseases, ultimately leading to improved health outcomes for both. By combining comparative structural and phylogenetic analysis, researchers can gain a more comprehensive understanding of *CXCR4* and its evolution, helping to inform the development of new treatments for diseases associated with this protein.

## Figures and Tables

**Table 1:** Gene ontology showing the molecular function of *CXCR4* protein.

GO-Term	Description	Network	Strength	FDR
GO:1990763	Arrestin family protein binding	2 of 10	2.55	0.0056
GO:0042379	Chemokine receptor binding	3 of 70	1.88	0.0052
GO:0045236	CXCR chemokine receptor binding	2 of 18	2.3	0.0127
GO:0019955	Cytokine binding	3 of 134	1.6	0.0127
GO:0004896	Cytokine receptor activity	3 of 97	1.74	0.0056
GO:0005126	Cytokine receptor binding	4 of 264	1.43	0.0052
GO:0019899	Enzyme binding	7 of 2239	0.75	0.0127
GO:0001664	G protein-coupled receptor binding	6 of 294	1.56	1.73 × 10^−5^
GO:0042289	MHC class II protein binding	2 of 6	2.77	0.0052
GO:0005515	Protein binding	10 of 7026	0.4	0.0477
GO:0044877	Protein-containing complex binding	7 of 1216	1.01	0.001
GO:0005102	Signaling receptor binding	9 of 1581	1.01	1.73 × 10^−5^
GO:0031702	Type 1 angiotensin receptor binding	2 of 7	2.71	0.0052
GO:0031826	Type 2a serotonin receptor binding	2 of 3	3.07	0.0023

## Data Availability

Not applicable.

## References

[1] Palomino D.C.T., Marti L.C. (2015). Chemokines and immunity. Einstein.

[2] Koizumi K., Hojo S., Akashi T., Yasumoto K., Saiki I. (2007). Chemokine receptors in cancer metastasis and cancer cell-derived chemokines in host immune response. Cancer Sci..

[3] Cravens P.D., Lipsky P.E. (2002). Dendritic cells, chemokine receptors and autoimmune inflammatory diseases. Immunol. Cell Biol..

[4] Bernardini G., Antonangeli F., Bonanni V., Santoni A. (2016). Dysregulation of chemokine/chemokine receptor axes and NK cell tissue localization during diseases. Front. Immunol..

[5] Zlotnik A., Yoshie O. (2000). Chemokines: A new classification system and their role in immunity. Immunity.

[6] Laing K.J., Secombes C.J. (2004). Chemokines. Dev. Comp. Immunol..

[7] Latek D., Modzelewska A., Trzaskowski B., Palczewski K., Filipek S. (2012). G protein-coupled receptors—Recent advances. Acta Biochim. Pol..

[8] Mollica Poeta V., Massara M., Capucetti A., Bonecchi R. (2019). Chemokines and chemokine receptors: New targets for cancer immunotherapy. Front. Immunol..

[9] Arimont M., Sun S.-L., Leurs R., Smit M., de Esch I.J., de Graaf C. (2017). Structural analysis of chemokine receptor-ligand interactions. J. Med. Chem..

[10] Fredriksson R., Lagerström M.C., Lundin L.-G., Schiöth H.B. (2003). The G-protein-coupled receptors in the human genome form five main families. Phylogenetic analysis, paralogon groups, and fingerprints. Mol. Pharmacol..

[11] Nibbs R.J., Graham G.J. (2013). Immune regulation by atypical chemokine receptors. Nat. Rev. Immunol..

[12] Luster A.D., Alon R., von Andrian U.H. (2005). Immune cell migration in inflammation: Present and future therapeutic targets. Nat. Immunol..

[13] Brauner-Osborne H., Wellendorph P., Jensen A.A. (2007). Structure, pharmacology and therapeutic prospects of family C G-protein coupled receptors. Curr. Drug Targets.

[14] Becker O.M., Marantz Y., Shacham S., Inbal B., Heifetz A., Kalid O., Bar-Haim S., Warshaviak D., Fichman M., Noiman S. (2004). G protein-coupled receptors: In silico drug discovery in 3D. Proc. Natl. Acad. Sci. USA.

[15] Lahti J.L., Tang G.W., Capriotti E., Liu T., Altman R.B. (2012). Bioinformatics and variability in drug response: A protein structural perspective. J. R. Soc. Interface.

[16] Day P.W., Rasmussen S.G., Parnot C., Fung J.J., Masood A., Kobilka T.S., Yao X.-J., Choi H.-J., Weis W.I., Rohrer D.K. (2007). A monoclonal antibody for G protein-coupled receptor crystallography. Nat. Methods.

[17] Wess J., Han S.-J., Kim S.-K., Jacobson K.A., Li J.H. (2008). Conformational changes involved in G-protein-coupled-receptor activation. Trends Pharmacol. Sci..

[18] Liu K., Wu L., Yuan S., Wu M., Xu Y., Sun Q., Li S., Zhao S., Hua T., Liu Z.-J. (2020). Structural basis of CXC chemokine receptor 2 activation and signalling. Nature.

[19] Ito S., Sato T., Maeta T. (2021). Role and therapeutic targeting of SDF-1α/CXCR4 axis in multiple myeloma. Cancers.

[20] Hara T., Tanegashima K. (2014). CXCL14 antagonizes the CXCL12-CXCR4 signaling axis. Biomol. Concepts.

[21] Bajoghli B. (2013). Evolution and function of chemokine receptors in the immune system of lower vertebrates. Eur. J. Immunol..

[22] Zlotnik A., Yoshie O. (2012). The chemokine superfamily revisited. Immunity.

[23] Zlotnik A., Yoshie O., Nomiyama H. (2006). The chemokine and chemokine receptor superfamilies and their molecular evolution. Genome Biol..

[24] Rajasekaran D., Fan C., Meng W., Pflugrath J.W., Lolis E.J. (2014). Structural insight into the evolution of a new chemokine family from zebrafish. Proteins: Struct. Funct. Bioinform..

[25] Haskill S., Peace A., Morris J., Sporn S.A., Anisowicz A., Lee S.W., Smith T., Martin G., Ralph P., Sager R. (1990). Identification of three related human GRO genes encoding cytokine functions. Proc. Natl. Acad. Sci. USA.

[26] Richmond A., Yang J., Su Y. (2009). The good and the bad of chemokines/chemokine receptors in melanoma. Pigment Cell Melanoma Res..

[27] Wu J., Chen Z.J. (2014). Innate immune sensing and signaling of cytosolic nucleic acids. Annu. Rev. Immunol..

[28] Yates A.D., Achuthan P., Akanni W., Allen J., Allen J., Alvarez-Jarreta J., Amode M.R., Armean I.M., Azov A.G., Bennett R. (2020). Ensembl 2020. Nucleic Acids Res..

[29] Sayers E.W., Cavanaugh M., Clark K., Pruitt K.D., Schoch C.L., Sherry S.T., Karsch-Mizrachi I. (2022). GenBank. Nucleic Acids Res..

[30] Yen Y.-C., Schafer C.T., Gustavsson M., Eberle S.A., Dominik P.K., Deneka D., Zhang P., Schall T.J., Kossiakoff A.A., Tesmer J.J. (2022). Structures of atypical chemokine receptor 3 reveal the basis for its promiscuity and signaling bias. Sci. Adv..

[31] Rodríguez D., Gutiérrez-de-Terán H. (2012). Characterization of the homodimerization interface and functional hotspots of the CXCR4 chemokine receptor. Proteins Struct. Funct. Bioinform..

[32] Culhane A.C., Schwarzl T., Sultana R., Picard K.C., Picard S.C., Lu T.H., Franklin K.R., French S.J., Papenhausen G., Correll M. (2010). GeneSigDB—A curated database of gene expression signatures. Nucleic Acids Res..

[33] Tatusova T., DiCuccio M., Badretdin A., Chetvernin V., Nawrocki E.P., Zaslavsky L., Lomsadze A., Pruitt K.D., Borodovsky M., Ostell J. (2016). NCBI prokaryotic genome annotation pipeline. Nucleic Acids Res..

[34] Burley S.K., Berman H.M., Kleywegt G.J., Markley J.L., Nakamura H., Velankar S. (2017). Protein Data Bank (PDB): The single global macromolecular structure archive. Protein Crystallography: Methods and Protocols.

[35] Kumar S., Stecher G., Li M., Knyaz C., Tamura K. (2018). MEGA X: Molecular evolutionary genetics analysis across computing platforms. Mol. Biol. Evol..

[36] Whelan S., Goldman N. (2001). A general empirical model of protein evolution derived from multiple protein families using a maximum-likelihood approach. Mol. Biol. Evol..

[37] Vilella A.J., Severin J., Ureta-Vidal A., Heng L., Durbin R., Birney E. (2009). EnsemblCompara GeneTrees: Complete, duplication-aware phylogenetic trees in vertebrates. Genome Res..

[38] Ahmad H.I., Khan F.A., Khan M.A., Imran S., Akhtar R.W., Pandupuspitasari N.S., Negara W., Chen J. (2022). Molecular Evolution of the Bactericidal/Permeability-Increasing Protein (BPIFA1) Regulating the Innate Immune Responses in Mammals. Genes.

[39] Guindon S., Gascuel O. (2003). A simple, fast, and accurate algorithm to estimate large phylogenies by maximum likelihood. Syst. Biol..

[40] Rokas A. (2011). Phylogenetic analysis of protein sequence data using the Randomized Axelerated Maximum Likelihood (RAXML) Program. Curr. Protoc. Mol. Biol..

[41] Edgar R.C. (2004). MUSCLE: Multiple sequence alignment with high accuracy and high throughput. Nucleic Acids Res..

[42] Posada D., Buckley T.R. (2004). Model selection and model averaging in phylogenetics: Advantages of Akaike information criterion and Bayesian approaches over likelihood ratio tests. Syst. Biol..

[43] Anisimova M., Gascuel O. (2006). Approximate likelihood-ratio test for branches: A fast, accurate, and powerful alternative. Syst. Biol..

[44] Conant G.C., Wagner G.P., Stadler P.F. (2007). Modeling amino acid substitution patterns in orthologous and paralogous genes. Mol. Phylogenetics Evol..

[45] Banerjee S. (2021). Computational Evolutionary Biology. Advances in Bioinformatics.

[46] Tamura K., Battistuzzi F.U., Billing-Ross P., Murillo O., Filipski A., Kumar S. (2012). Estimating divergence times in large molecular phylogenies. Proc. Natl. Acad. Sci. USA.

[47] Kelley L.A., Mezulis S., Yates C.M., Wass M.N., Sternberg M.J. (2015). The Phyre2 web portal for protein modeling, prediction and analysis. Nat. Protoc..

[48] Schwede T., Kopp J., Guex N., Peitsch M.C. (2003). SWISS-MODEL: An automated protein homology-modeling server. Nucleic Acids Res..

[49] Yang J., Zhang Y. (2015). I-TASSER server: New development for protein structure and function predictions. Nucleic Acids Res..

[50] Pettersen E.F., Goddard T.D., Huang C.C., Couch G.S., Greenblatt D.M., Meng E.C., Ferrin T.E. (2004). UCSF Chimera—A visualization system for exploratory research and analysis. J. Comput. Chem..

[51] Ahmad H.I., Asif A.R., Ahmad M.J., Jabbir F., Adnan M., Ahmed S., Afzal G., Saleem A.H., Li L., Jiang H. (2020). Adaptive evolution of peptidoglycan recognition protein family regulates the innate signaling against microbial pathogens in vertebrates. Microb. Pathog..

[52] Wiederstein M., Sippl M.J. (2007). ProSA-web: Interactive web service for the recognition of errors in three-dimensional structures of proteins. Nucleic Acids Res..

[53] Ahmad H.I., Majeed M.B.B., Ahmad M.Z., Jabbar A., Maqbool B., Ahmed S., Mustafa H., Simirgiotis M.J., Chen J. (2021). Comparative analysis of the mitochondrial proteins reveals complex structural and functional relationships in Fasciola species. Microb. Pathog..

[54] Cheng J., Zhen Y., Miksys S., Beyoğlu D., Krausz K.W., Tyndale R.F., Yu A., Idle J.R., Gonzalez F.J. (2013). Potential role of CYP2D6 in the central nervous system. Xenobiotica.

[55] Källberg M., Margaryan G., Wang S., Ma J., Xu J. (2014). RaptorX server: A resource for template-based protein structure modeling. Protein Structure Prediction.

[56] Wu Q., Peng Z., Zhang Y., Yang J. (2018). COACH-D: Improved protein-ligand binding sites prediction with refined ligand-binding poses through molecular docking. Nucleic Acids Res..

[57] Yang J., Roy A., Zhang Y. (2012). BioLiP: A semi-manually curated database for biologically relevant ligand-protein interactions. Nucleic Acids Res..

[58] Ngan C.-H., Hall D.R., Zerbe B., Grove L.E., Kozakov D., Vajda S. (2012). FTSite: High accuracy detection of ligand binding sites on unbound protein structures. Bioinformatics.

[59] Kozakov D., Grove L.E., Hall D.R., Bohnuud T., Mottarella S.E., Luo L., Xia B., Beglov D., Vajda S. (2015). The FTMap family of web servers for determining and characterizing ligand-binding hot spots of proteins. Nat. Protoc..

[60] Ahmad H.I., Iqbal A., Ijaz N., Ullah M.I., Asif A.R., Rahman A., Mehmood T., Haider G., Ahmed S., Mahmoud S.F. (2022). Molecular Evolution of the Activating Transcription Factors Shapes the Adaptive Cellular Responses to Oxidative Stress. Oxidative Med. Cell. Longev..

[61] Singh H., Srivastava H.K., Raghava G.P. (2016). A web server for analysis, comparison and prediction of protein ligand binding sites. Biol. Direct.

[62] Ahmad H.I., Afzal G., Jamal A., Kiran S., Khan M.A., Mehmood K., Kamran Z., Ahmed I., Ahmad S., Ahmad A. (2021). In silico structural, functional, and phylogenetic analysis of cytochrome (CYPD) protein family. BioMed Res. Int..

[63] Von Mering C., Jensen L.J., Kuhn M., Chaffron S., Doerks T., Krüger B., Snel B., Bork P. (2007). STRING 7—Recent developments in the integration and prediction of protein interactions. Nucleic Acids Res..

[64] Kohl M., Wiese S., Warscheid B. (2011). Cytoscape: Software for visualization and analysis of biological networks. Data Mining in Proteomics.

[65] Szklarczyk D., Franceschini A., Wyder S., Forslund K., Heller D., Huerta-Cepas J., Simonovic M., Roth A., Santos A., Tsafou K.P. (2015). STRING v10: Protein–protein interaction networks, integrated over the tree of life. Nucleic Acids Res..

[66] Gouet P., Courcelle E. (2002). ENDscript: A workflow to display sequence and structure information. Bioinformatics.

[67] Buchan D.W., Jones D.T. (2019). The PSIPRED protein analysis workbench: 20 years on. Nucleic Acids Res..

[68] Jabbir F., Irfan M., Mustafa G., Ahmad H.I. (2019). Bioinformatics approaches to explore the phylogeny and role of BRCA1 in breast cancer. Crit. Rev. Eukaryot. Gene Expr..

[69] Mizoguchi T., Verkade H., Heath J.K., Kuroiwa A., Kikuchi Y. (2008). Sdf1/Cxcr4 signaling controls the dorsal migration of endodermal cells during zebrafish gastrulation. Development.

[70] Tulotta C., Stefanescu C., Chen Q., Torraca V., Meijer A., Snaar-Jagalska B. (2019). CXCR4 signaling regulates metastatic onset by controlling neutrophil motility and response to malignant cells. Sci. Rep..

[71] Beutler B., Rehli M. (2002). Evolution of the TIR, tolls and TLRs: Functional inferences from computational biology. Toll-Like Receptor Family Members and Their Ligands.

[72] DeVries M.E., Kelvin A.A., Xu L., Ran L., Robinson J., Kelvin D.J. (2006). Defining the origins and evolution of the chemokine/chemokine receptor system. J. Immunol..

[73] Murali S., Aradhyam G.K. (2023). Structure-function relationship and physiological role of apelin and its G protein coupled receptor. Biophys. Rev..

[74] Liggett S.B. (2011). Phosphorylation barcoding as a mechanism of directing GPCR signaling. Sci. Signal..

[75] Onuffer J.J., Horuk R. (2002). Chemokines, chemokine receptors and small-molecule antagonists: Recent developments. Trends Pharmacol. Sci..

[76] Nemoto W., Toh H. (2006). Membrane interactive α-helices in GPCRs as a novel drug target. Curr. Protein Pept. Sci..

[77] Kwon H.R. (2010). Study of the Structure and Function of CXC Chemokine Receptor 2. Ph.D. Thesis.

[78] Nasser M.W., Raghuwanshi S.K., Malloy K.M., Gangavarapu P., Shim J.-Y., Rajarathnam K., Richardson R.M. (2007). CXCR1 and CXCR2 activation and regulation: Role of aspartate 199 of the second extracellular loop of CXCR2 in CXCL8-mediated rapid receptor internalization. J. Biol. Chem..

[79] Peeters M., van Westen G., Li Q., IJzerman A. (2011). Importance of the extracellular loops in G protein-coupled receptors for ligand recognition and receptor activation. Trends Pharmacol. Sci..

[80] Hoare S.R. (2005). Mechanisms of peptide and nonpeptide ligand binding to Class B G-protein-coupled receptors. Drug Discov. Today.

[81] Bondos S.E., Dunker A.K., Uversky V.N. (2022). Intrinsically disordered proteins play diverse roles in cell signaling. Cell Commun. Signal..

